# Public health implications of plasmid-mediated quinolone and aminoglycoside resistance genes in *Escherichia coli* inhabiting a major anthropogenic river of India

**DOI:** 10.1017/S095026882200053X

**Published:** 2022-03-28

**Authors:** Nambram Somendro Singh, Neelja Singhal, Manish Kumar, Jugsharan Singh Virdi

**Affiliations:** 1 Department of Microbiology, University of Delhi South Campus, New Delhi, India; 2 Department of Biophysics, University of Delhi South Campus, New Delhi, India

**Keywords:** Conjugation, horizontal gene transfer, plasmid-mediated aminoglycoside resistance genes, plasmid-mediated quinolone resistance genes

## Abstract

Presence of antimicrobial resistance (AMR) genes in *Escherichia coli* inhabiting anthropogenic rivers is an important public health concern because plasmid-mediated AMR genes can easily spread to other pathogens by horizontal gene transfer. Besides *β*-lactams, quinolones and aminoglycosides are the major antibiotics against *E. coli.* In the present study, we have investigated the presence of plasmid-mediated quinolone resistance (PMQR) and aminoglycoside resistance genes in *E. coli* isolated from a major river of northern India. Our results revealed that majority of the strains were phenotypically susceptible for fluoroquinolones and some aminoglycosides like amikacin, netilmicin, tobramycin and gentamicin. However, 16.39% of the strains were resistant for streptomycin, 8.19% for kanamycin and 3.30% for gentamicin. Of the various PMQR genes investigated, only *qnrS1* was present in 24.59% of the strains along with IS*Ecl2*. Aminoglycoside-resistance genes like *strA-strB* were found to be present in 16.39%, *aphA1* in 8.19% and *aacC*2 in only 3.30% of the strains. Though, no co-relation was observed between phenotypic resistance for fluorquinolones and presence of PMQR genes, phenotypic resistance for streptomycin, kanamycin and gentamicin exactly co-related with the presence of the genes *strA-strB*, *aphA1* and *aacC2*, respectively. Moreover, all the AMR genes discerned in aquatic *E. coli* were found to be situated on conjugative plasmids and, thus easily transferrable. Our study accentuates the importance of routine surveillance of urban rivers to curtail the spread of AMR genes in aquatic pathogens.

## Introduction

*Escherichia coli* is a diverse bacterial species, strains of which might be commensal or pathogenic in nature. It is primarily an inhabitant of the lower intestinal tract of humans and warm-blooded animals and is discharged in the environment through faeces and wastewater treatment plants [1]. Among all the members of the family *Enterobacteriaceae*, *E. coli* has a remarkable capability to serve as a donor and recipient of antimicrobial resistance (AMR) genes. It is therefore regarded as a major reservoir of AMR genes which can be disseminated to other bacteria by horizontal gene transfer. Thus, AMR in *E. coli* is considered as one of the major challenges in both humans and animals, worldwide [2–4].

Besides *β*-lactams, quinolones and aminoglycosides are the major antibiotics which are used for treatment of infections caused by *E. coli*. Quinolones target the bacterial DNA gyrase or the topoisomerase IV enzyme, thereby inhibiting DNA replication and transcription. In *E. coli*, mutations in the quinolone resistance-determining region of the chromosomal DNA gyrase and DNA topoisomerase IV are regarded as an important mechanism underlying fluoroquinolone resistance [5, 6]. Besides chromosomal gene mutations, several plasmid encoded resistance mechanisms have been identified in *E. coli* like, (i) genes encoding pentapeptide repeat family proteins which protect bacterial DNA gyrase and topoisomerase IV from inhibitory effect of quinolones (*qnrA to qnrD* and *qnrS*), (ii) *aac(6′)-Ib-cr* which encodes actyltransferases that modify fluoroquinolones like ciprofloxacin and ofloxacin, and (iii) genes encoding efflux pump proteins like *qepA* and *oqxAB* [7]. Since the probability of spontaneous multiple mutations is quite less (10^−14^ to 10^−16^ for fluoroquinolones) the plasmid-mediated quinolone resistance (PMQR) determinants might play a major role in enabling bacterial survival in the presence of quinolones [8]. A research study indicated that although the PMQR genes cannot confer a high level of resistance for quinolones and fluoroquinolones, they might reduce the susceptibility of *E. coli* for these antibiotics [9]. Several researchers have reported that insertion sequences (IS) play an important role in the mobilisation of PMQR genes [10, 11]. IS like IS*Ecl2* and IS*26* have been associated with the mobilisation of PMQR genes like *qnrS* and *aac(6′)-Ib-cr*, respectively [11]. However, most of the studies regarding PMQR genes and their genetic environment have been conducted on clinical isolates [10–12] and only a few studies have been conducted on aquatic *E. coli* [13–15].

Aminoglycosides bind to the 30S ribosomal subunit and interfere with the bacterial protein synthesis. In *E. coli*, resistance to aminoglycosides can develop by mutations in the 16S rRNA, modification of the drug target(s) or by enzymatic inactivation of aminoglycosides by the bacterial enzymes. Modification of the aminoglycoside target site is performed by 16S rRNA methylases like ArmA, RmtA to RmtH and NmpA which methylate some amino acids of the 16S rRNA, resulting in resistance for amikacin, gentamicin, tobramycin and netilmicin [16]. In *E. coli*, three types of aminoglycoside modifying enzymes are known *viz.*, phosphotransferases, acetyltransferases and nucleotidyltransferases. Genes encoding these enzymes are frequently located on plasmids facilitating their transfer to other pathogens. The linked *strA-strB* genes which encode for aminoglycoside phosphotransferases are the most common streptomycin resistance genes prevalent in *E. coli*, worldwide. In *E. coli*, phosphotransferase gene *aphA1* which confers kanamycin resistance and *aacC2* (an acetyltransferase) that confers gentamicin resistance are also reported [17, 18].

Most of the studies regarding distribution and characterisation AMR genes have been conducted on clinical isolates of *E. coli*. Studies regarding the prevalence and characterisation of AMR and virulence genes in *E. coli* inhabiting natural water bodies, especially urban rivers, are quite less [19–25]. It is important to study the AMR genes of *E. coli*, a prominent faecal indicator bacteria residing in urban rivers because these rivers are impacted by various anthropogenic activities. Thus, urban rivers represent a reservoir of diverse *E. coli* and an ideal ecosystem for transfer and dissemination of AMR genes from aquatic *E. coli* to other pathogens [26–30]. In an earlier study, we had reported the distribution and characteristics of *β*-lactamase genes of *E. coli* isolated from Yamuna, a prominent river of northern India [31]. A collection of 61 strains representing the four phylogroups of *E. coli viz.* A, B1 (non-pathogenic phylogroups) and B2, D (pathogenic phylogroups) was investigated [31]. Here, we have studied the phenotypic susceptibilities of these *E. coli* strains for quinolones and aminoglycosides, and the presence of PMQR and plasmid-mediated aminoglycoside resistance genes. We have also tried to discern a correlation between AMR genes and phylogroups, if any. The genetic elements associated with the PMQR genes were also studied to understand their role in the spread of PMQR genes in the environment.

## Materials and methods

### Bacterial strains

A total of 61 strains of *E. coli* collected from various sites along the entire stretch of river Yamuna across the National Capital Region of India were used in this study. These strains were transported to the laboratory on ice in sterile screw-capped bottles and processed within 6 h. The details of the sampling sites, year of isolation, seasonality and the methods used for enrichment and isolation of *E. coli* have been described in detail, previously [31]. All the strains were confirmed by biochemical testing and sequencing of the gene encoding 16S rDNA. The strains were serotyped at the National *Salmonella* and *Escherichia* Centre, Central Research Institute, Kasauli, Himachal Pradesh, India. The phylogenetic profiles of the strains were determined by triplex PCR which revealed that these strains belonged to the phylogroups A, B1, B2 and D [32]. The azide-resistant *E. coli* strain J53 used as the recipient during conjugation experiments was a gift from Dr George A. Jacoby and was provided to us by Dr Sulagna Basu (National Institute of Cholera and Enteric Diseases, Kolkata, India).

### Antimicrobial susceptibility testing for quinolones and aminoglycosides

Antimicrobial susceptibilities of all the 61 *E. coli* strains for quinolones and aminoglycosides were determined by Kirby-Bauer disk diffusion test, using antibiotic disks (Himedia, Mumbai, India) and following the guidelines of Clinical Laboratory Standards Institute [33]. The antibiotic disks which were used in this study (charge in μg/disk) included nalidixic acid (30 μg), ciprofloxacin (5 μg) and ofloxacin (5 μg) for quinolone susceptibility, and streptomycin (10 μg), kanamycin (30 μg), tobramycin (10 μg), netilmycin (30 μg) and amikacin (30 μg) for aminoglycoside susceptibility.

### Detection of PMQR genes

For PCR-based detection of PMQR genes, *qnrA*, *qnrB*, *qnrC*, *qnrD*, *qnrS*, *qepA*, *oqxA*, *oqxB* and *aac(6′)-Ib-cr*, DNA of the *E. coli* strains was isolated by boiling lysis procedure [34]. The 25 μl PCR-reaction mixture contained 2.5 μl of 1× buffer, 200 μM of each dNTP, 20 pmol of the forward and reverse primers, 1 U of Taq DNA polymerase and 10 μl of template DNA. PCR was carried out in My Cycler^™^ Thermal Cycler, using the published primers and PCR conditions (Table 1). PCR amplicons were purified and sequenced using the methods described earlier [35]. Briefly, PCR amplicons were visualised after electrophoresis on 1% agarose gels at 80 V and purified using Hi-Yield^™^ extraction kit (RBC Bioscience, New Taipei City, Taiwan) following the manufacturer's protocol. The purified amplicons were submitted for sequencing to a commercial facility (Invitrogen BioServices India Pvt. Ltd., Bangalore, India) where they were sequenced using Sanger's method. Similarity search of the nucleotide sequences was performed using NCBI-BLASTn.

**Table 1.** Details of primers and PCR conditions used for analyses of plasmid-associated quinolone and aminoglycoside resistance genes, and genetic environment of *qnrS* in *E. coli* isolated from a major urban river of India

Primers	Nucleotide sequence	Target genes	Amplicon size (bp)	Annealing temperature (°C)	References
RMTA-F RMTA-R	5′-CTAGCGTCCATCCTTTCCTC-3′ 5′-TTTGCTTCCATGCCCTTGCC-3′	*rmtA*	653	57	[36]
RMTB-F RMTB-R	5′-GCTTTCTGCGGGCGATGTAA-3′ 5′-ATGCAATGCCGCGCTCGT AT-3′	*rmtB*	173	60	[36]
RMTC-F RMTC-R	5′-CGAAGAAGTAACAGCCAAAG-3′ 5′-ATCCCAACATCTCTCCCACT-3′	*rmtC*	711	55	[36]
ARMA-F ARMA-R	5′-ATTCTGCCTATCCTAATTGG-3′ 5′-ACC TATACTTTATCGTCGTC-3′	*armA*	315	46	[36]
str-F str-R	5′-TATCTGCGATTGGACCCTCTG-3′ 5′-CATTGCTCATCATTTGATCGGCT-3′	*strA-strB*	538	62	[54]
aacC2-F aacC2-R	5′-TAGAGGAGTATCGCGATGC-3′ 5′ATTATCATTGTCGACGGCCT-3′	*aacC2*	861	55	[18]
AphA1-F AphA1-R	5′-ATGGGCTCGCGATAATGTC-3′ 5′-CTCACCGAGGCAGTTCCAT-3′	*aphA1*	600	55	[17]
QA-F QA-R	5′-TCGCCGCTGCCGCTTTTAT-3′ 5′-TTCGAGGTTGACCCGTCTG-3′	*qnrA*	517	60	[55]
QB-F QB-R	5′-AACCTGAAAGATGCCATT-3′ 5′-AAGGCCTTGTAAATCAAC-3′	*qnrB*	405	50	[55]
QC-F QC-R	5′-GGGTTGTACATTTATTGAATC-3′ 5′-TCCACTTTACGAGGTTCT-3′	*qnrC*	447	50	[55]
QD-F QD-R	5′-CGAGATCAATTTACGGGGAATA-3′ 5′-CGAGATCAATTTACGGGGAATA-3′	*qnrD*	582	57	[56]
QS-F QS-R	5′-GACGTGCTAACTTGCGTGAT-3′ 5′-GATCTAAACCGTCGAGTTCG-3′	*qnrS*	456	55	[31]
ACC-F ACC-R	5′TTGCGATGCTCTATGAGTGGCTA-3′ 5′-CTCGAATGCCTGGCGTGTTT-3′	*aac(6′)-Ib*	482	60	[57]
QEP-F QEP-R	5′-CTGCAGGTACTGCGTCATG-3′ 5′-CGTGTTGCTGGAGTTCTTC-3′	*qepA*	403	55	[37]
XA-F XA-R	5′-GACAGCGTCGCACAGAATG-3′ 5′-GGAGACGAGGTTGGTATGGA-3′	*oxqA*	339	62	[57]
XB-F XB_R	5′-CGAAGAAAGACCTCCCTACCC-3′ 5′-CGCCGCCAATGAGATACA-3′	*oxqB*	240	62	[57]
Pre-qnrs1-F Pre-qnrs1-R	5′-CTGATAACACTTCAACCATC-3′ 5′-TCGTTTTATAAATTTGAGCG-3′	IS*Ecl2*, *qnrS1*	1113	50	[10]
IS26-F Pre-qnrs1-R	5′GTACGGCCCACAGAATGATGTC-3′ 5′-TCGTTTTATAAATTTGAGCG-3′	IS*26*, IS*Ecl2*, *qnrS1*	2381	50	[10]
IS26-F AAC-R	5′GTACGGCCCACAGAATGATGTC-3′ 5′-CTCGAATGCCTGGCGTGTTT-3′	IS*26*, *aac(6′)-Ib*	1100	45	[57]

### Analysis of the genetic environment of *qnrS*

Genetic environment of *qnrS* was analysed by PCR amplification using the published primers and PCR protocol [10]. Insertion sequences IS*26* and IS*Ecl2* were targeted in the upstream region of *qnrS* gene. The components of the PCR reaction mixture were the same as used for PCR amplification of PMQR genes. The PCR conditions and the details of the primers are mentioned in Table 1. The PCR amplicons were purified and sequenced as described earlier, using the respective forward primers. Similarity search was performed for the sequences obtained using NCBI-BLAST.

### Detection of plasmid-mediated aminoglycoside resistance genes

PCR-based detection of plasmid-mediated aminoglycoside resistance genes encoding 16S rRNA methylases – *armA*, *rmtA*, *rmtB*, *rmtC* and *rmtD* and aminoglycoside modifying enzymes – *aacC*, *strA-strB*, *aphA1*, *aphA2* was carried out using published primers [17, 36]. The components of the PCR reaction mixture and the PCR conditions were the same as for amplification of PMQR genes, except the annealing temperatures, which have been mentioned in Table 1. The amplicons were purified and sequenced as described earlier and the similarity search was performed using NCBI-BLASTn.

### Transferability of AMR genes by conjugation and plasmid analysis

To confirm if the PMQR and aminoglycoside resistance genes were transferrable, conjugal transfer of these genes was assessed with a broth culture mating assay using an azide-resistant *E. coli* J53 as recipient, as also described earlier [21]. Briefly, the donor and the recipient *E. coli* J53 strains were separately grown in LB broth at 37 °C, 200 rpm for 12–14 h. Conjugal transfer was carried by mixing each donor and recipient in a 1:1 ratio, followed by incubation at 37 °C for 12–14 h under static conditions. Subsequently, appropriate culture dilutions were spread plated on LB agar containing sodium azide (100 μg/ml) supplemented with ampicillin (100 μg/ml) and incubated at 37 °C for 16–18 h. The transferability of the PMQR (*qnrS1*, *aac(6′)-Ib*) and aminoglycoside resistance (*strA-strB*, *aphA1* and *aacC2*) genes was confirmed by PCR amplification of the plasmid DNA isolated from the transconjugants.

### Accession numbers

The partial coding DNA sequence (CDS) of *qnrS1* genes including their genetic environment were identical in all the 15 strains, therefore the DNA sequence of only one representative strain (KK16) was submitted to GenBank (NCBI) with the accession number MG434695. Similarly, the partial CDS of *strA-strB* gene of only one representative strain (ISF) was submitted to NCBI GenBank under the accession number MT995846.

## Results and discussion

### Quinolone susceptibilities and PMQR genes

The zone diameters (in mm) of the bacterial growth around antibiotic disks of nalidixic acid, ciprofloxacin and ofloxacin were ≥19, ≥31 and ≥16, respectively, indicating that all the *E. coli* strains were susceptible to these antibiotics, as also recommended by the CLSI 2018 guidelines [33]. However, the growth zone diameters (in mm) of two *E. coli* strains IP24 and IPE around the antibiotic disks of nalidixic acid, ciprofloxacin and ofloxacin ≤14, ≤20 and ≤12, respectively, indicate that both these strains were resistant for these antibiotics. The results of the antibiotic susceptibility testing are presented in Table 2. PCR-based testing revealed that none of the *E. coli* strains harboured the PMQR genes which encode for the pentapeptide repeat family proteins, *qnrA*, *qnrB*, *qnrC*, *qnrD* and *qepA*. Similarly, PMQR efflux genes like *oqxA* and *oqxB* were not detected in any strain. However, the PMQR gene *qnrS* was detected in 24.59% (*n* = 15) of the strains. The 456 bp amplicon of the *qnrS* gene of one representative strain was sequenced and similarity search by BLASTn revealed that it shared 99% homology with the *qnrS1* gene. Thus, it can be inferred that *qnrS1* was the predominant PMQR gene present in river Yamuna *E. coli* isolates. Earlier studies also reported that *qnrS* type genes were highly prevalent in *E. coli* isolated from the waterbodies of the world [37, 38].

**Table 2.** Resistance phenotypes and plasmid-associated genes encoding flouro(quinolone) and aminoglycoside resistance in *E. coli* strains isolated from a major urban river of India

Strain designation	Phylogroup	Antibiotic resistance	Resistance gene(s)	Genetic environment of *qnrS1*
IPH	A	–	–	–
IPS	A	–	–	–
IS5	A	–	–	–
IS57	A	–	–	–
IS58	A	STM	*strA-strB*	–
IS76	A	–	–	–
IPI	A	–	–	–
IP1	A	–	–	–
KK36	A	–	*qnrS1*	IS*Ecl2*
KK30	A	–	*qnrS1*	IS*Ecl2*
NG28	A	–	–	–
NG9	A	–	–	–
PA18	A	–	–	–
IP11	A	–	–	–
IP18	A	–	*qnrS1*	IS*Ecl2*
IS40	A	–	–	–
MKNJ	A	–	*qnrS1*	IS*Ecl2*
WB23	A	–	*qnrS1*	IS*Ecl2*
WB31	A	–	*qnrS1*	IS*Ecl2*
DND14	A	–	–	–
KP5S	A	–	–	–
KP21	A	–	*qnrS1*	IS*Ecl2*
IST	A	KAN	*qnrS1*, *aphA1*	IS*Ecl2*
NG3	A	–	–	–
PA21	A	–	–	–
WB28	A	GEN	*aacC*	–
IPG	A	–	–	–
IS54	A	–	–	–
IP24	B1	NAL, CIP, OFX	*qnrS1*	IS*Ecl2*
ISD	B1	–	*qnrS1*	IS*Ecl2*
KK32	B1	STM	*strA-strB*	–
NG29	B1	–	–	–
PA4	B1	–	–	–
MKNE	B1	–	–	–
SVI	B1	–	–	–
DND6	B1	–	–	–
DND1	B1	–	–	–
WB4	B1	–	–	–
NG32	B1	–	–	–
IP5N	B1	–	–	–
PA32	B1	–	–	–
KK46	B1	–	–	–
WB14	B1	–	–	–
ISI	B2	–	–	–
KP24	B2	STM	*strA-strB*	–
MKNG	B2	–	–	–
IP9	D	–	–	–
IPE	D	NAL, CIP, OFX, KAN	*aaC(6′)-Ib-cr*, *aphA1*	–
ISE	D	STM	*strA-strB*	–
KK16	D	STM	*qnrS1*, *strA-strB*	IS*Ecl2*
KK45	D	KAN, GEN	*aphA1*, *aacC*	–
NG37	D	STM, KAN	*strA-strB*, *aphA1*	–
PA6	D	–	–	–
KP31	D	–	–	–
MKND	D	KAN	*aphA1*	–
PA24	D	–	–	–
WB6	D	STM	*qnrS1*, *strA-strB*	IS*Ecl2*
KK38	D	STM	*strA-strB*	–
KK31	D	–	*qnrS1*	IS*Ecl2*
KKA	D	STM	*qnrS1*, *strA-strB*	IS*Ecl2*
PA12	D	STM	*qnrS1*, *strA-strB*	IS*Ecl2*

STM, streptomycin; GEN, gentamicin; KAN, kanamycin; NAL, nalidixic acid; CIP, ciprofloxacin; OFX, ofloxacin.

The PMQR gene *aac(6′)-Ib-cr* was detected in only one *E. coli* strain (IPE). The *aac(6′)-Ib-cr* gene encodes for a common aminoglycoside acetyltransferase AAC(6′)-Ib-cr which is involved in acetylation of quinolones. Earlier studies have also reported that *qnrS* and *aac(6′)-Ib-cr* were the most frequent PMQR genes in *E. coli* isolated from environmental sources, worldwide [14, 39, 40]. The PMQR gene *qnrS1* was present in strains of both pathogenic and non-pathogenic phylogroups.

Phenotypic resistance to quinolones did not co-relate with the presence of the PMQR genes because several strains of *E. coli* which were phenotypically susceptible to flouoroquinolones harboured the *qnrS1* gene. An earlier study reported that PMQR genes provide a low-level of resistance for flouoroquinolones which though usually do not surpass the clinical breakpoints; they make the treatment difficult [9]. This suggests that clinical breakpoints should be reassessed in the context of PMQR genes, which results in reduced susceptibility and consequent therapeutic failures, despite going undetected by traditional phenotypic methods [41]. Thus, phenotypic methods for testing flouoroquinolone susceptibilities might result in underestimation of prevalence of quinolone resistance, and confirmation of PMQR genes by PCR is necessary to assess the true flouoroquinolone susceptibilities of *E. coli*. Moreover, due to the presence of PMQR genes in aquatic *E. coli*, these could serve as potential reservoirs for undetected spread and dissemination of PMQR genes to other waterborne pathogens.

### Genetic environment associated with *qnrS1* and *aac(6′)-Ib-cr* genes

PCR mapping was used to identify the genetic structures associated with *qnrS1* in all the *qnrS1*-positive *E. coli* strains. PCR amplification resulted in 1113 bp amplicon in each strain. Similarity search revealed that in all the strains, IS*Ecl2* was present at 303 bp upstream of the start codon of *qnrS1* (Table 2). The insertion sequence IS*Ecl2* belongs to the IS*3* family of insertion sequences. The genetic environment associated with *qnrS1* was similar to that reported for *E. coli* isolated from Vietnam and France [10, 42]. However, the role of IS*Ecl2* in the mobilisation of *qnrS1* gene is not known, so far. The PCR mapping from earlier studies suggested that mobilisation of *qnrS1* gene might have occurred as an independent event [11].

The genetic elements surrounding the *aac(6′)-Ib-cr* gene detected in the *E. coli* strain IPE were also investigated by PCR mapping and similarity search at NCBI. None of the IS, particularly the IS*26* which has been widely reported to be present upstream of *aac(6′)-Ib-cr*, was found in the *aac(6′)-Ib-cr*-positive *E. coli* strain of river Yamuna [11].

### Aminoglycoside susceptibilities and plasmid-mediated aminoglycoside resistance genes

The bacterial growth zone diameters (in mm) around the antibiotic disks of amikacin, netilmicin and tobramycin were ≥17, ≥15 and ≥15, respectively. Thus, all the *E. coli* strains were considered phenotypically susceptible for amikacin, netilmicin and tobramycin as also recommended by the CLSI 2018 guidelines [33]. However, zone diameters of 16.39% (*n* = 10) of the *E. coli* strains around the antibiotic disks of streptomycin were ≤11, of 8.19% (*n* = 5) strains around kanamycin disks were ≤13 and of 3.30% (*n* = 2) of the strains around gentamicin disks were ≤ 12 indicating that these strains were resistant for these antibiotics. The results of the antibiotic susceptibility testing are presented in Table 2. Thus, our results are similar to an earlier study which also reported that *E. coli* isolated from waterbodies of Malaysia exhibited lower levels of resistance for aminoglycosides [43]. However, another study from India reported that *E. coli* strains isolated from coastal waters of India were highly resistant for streptomycin and gentamicin [44].

Though 16S rRNA methylase genes have been reported in clinical strains [45], very few studies have reported the distribution of these genes in aquatic strains of *E. coli* [46]. Our results revealed that plasmid-mediated 16S rRNA methylase genes (*armA*, *rmtA*, *rmtB*, *rmtC* and *rmtD*) were not present in *E. coli* strains of river Yamuna. It has been proposed that rRNA methylases that confer resistance to aminoglycosides have not disseminated widely in *E. coli* for reasons related to fitness [47, 48]. The linked *strA*-*strB* genes which encode for phosphotransferases and are reportedly the most prevalent streptomycin resistance genes in *E. coli* worldwide [49] were found to be present in only 16.39% (*n* = 10) of the *E. coli* isolates (Table 2). A study from Capetown, South Africa also reported a high prevalence of *strA-strB* in *E. coli* strains isolated from wastewater effluents [50]. The aminoglycoside phosphotransferase gene *aphA1* which confers resistance to kanamycin was present in 8.19% (*n* = 5) of the strains. The aminoglycoside acetyltransferase gene *aacC2* that confers resistance to gentamicin was present in only 3.3% (*n* = 2) of the strains (Table 2). Notably, unlike the quinolones, phenotypic resistance to a particular aminoglycoside antibiotic exactly co-related with the presence of its corresponding resistance gene. It has been reported that genes encoding aminoglycoside-modifying enzymes have disseminated globally [49]. This might be due to the fact that these genes are frequently found on transposons, which might have played an important role in the dissemination of aminoglycoside resistance across inter- and intra-species boundaries [51]. Interestingly, the aminoglycoside phosphotransferases *strA-strB* and *aphA1* were more prevalent in strains of the pathogenic phylogroup D, than in the strains of the non-pathogenic phylogroups (Table 2).

Co-occurrence of fluoroquinolone and aminoglycoside resistance genes was observed in only six *E. coli* strains, *viz*. KK16, WB6, KKA, PA12, IST and IPE.

### Analysis of the transconjugants

Analysis of the plasmid DNA isolated from the transconjugants revealed that *qnrs1* in the 14 strains and *aac(6′)-Ib* detected in one *E. coli* strain were plasmid-mediated and transferrable. Similarly, *strA-strB*, *aphA1* and *aacC*2 genes were also found to be plasmid-mediated and transferrable. Previous studies have indicated that conjugative plasmids were highly transferable and played a key role in conferring a multi-resistance phenotype to waterborne *E. coli* [14, 20, 52, 53].

## Conclusion

Of the various PMQR genes investigated, *qnrS1* was present in 25% of the strains, and IS*Ecl2* was present in its upstream region. Among the aminoglycoside resistance genes, genes encoding for *strA-strB* and *aphA1* were present in 16.39% and 8.19% of the *E. coli* strains, respectively. Though no co-relation was observed between phenotypic resistance for quinolones and PMQR genes, phenotypic resistance for streptomycin, kanamycin and gentamicin co-related well with the presence of plasmid-mediated aminoglycoside resistance genes *strA-strB*, *aphA1* and *aacC2*, respectively. Since PMQR and aminoglycoside resistance genes were situated on conjugative plasmids they could be easily disseminated to other pathogens. Thus, our study highlights the importance of routine surveillance of microbial population of urban waterbodies to check the wide spread dissemination of antibiotic resistance determinants.

## Data Availability

Not applicable.
